# Evaluation of cellular water exchange in a mouse glioma model using dynamic contrast-enhanced MRI with two flip angles

**DOI:** 10.1038/s41598-023-29991-1

**Published:** 2023-02-21

**Authors:** Karl Kiser, Jin Zhang, Ayesha Bharadwaj Das, James A. Tranos, Youssef Zaim Wadghiri, Sungheon Gene Kim

**Affiliations:** 1 Department of Radiology, Weill Cornell Medical College, 1300 York Avenue, New York, NY 10065 WMC Box 141, USA; 2 Center for Biomedical Imaging (CBI), Center for Advanced Imaging Innovation and Research (CAI2R), Department of Radiology, NYU Grossman School of Medicine, New York, NY USA

**Keywords:** Prognostic markers, Preclinical research, Cancer imaging, Cancer metabolism, Cancer microenvironment, Tumour biomarkers, Tumour heterogeneity, Cancer therapy

## Abstract

This manuscript aims to evaluate the robustness and significance of the water efflux rate constant (*k*_*io*_) parameter estimated using the two flip-angle Dynamic Contrast-Enhanced (DCE) MRI approach with a murine glioblastoma model at 7 T. The repeatability of contrast kinetic parameters and *k*_*io*_ measurement was assessed by a test–retest experiment (n = 7). The association of *k*_*io*_ with cellular metabolism was investigated through DCE-MRI and FDG-PET experiments (n = 7). Tumor response to a combination therapy of bevacizumab and fluorouracil (5FU) monitored by contrast kinetic parameters and *k*_*io*_ (n = 10). Test–retest experiments demonstrated compartmental volume fractions (*v*_*e*_ and *v*_*p*_) remained consistent between scans while the vascular functional measures (*F*_*p*_ and *PS)* and *k*_*io*_ showed noticeable changes, most likely due to physiological changes of the tumor. The standardized uptake value (SUV) of tumors has a linear correlation with *k*_*io*_ (R^2^ = 0.547), a positive correlation with *F*_*p*_ (R^2^ = 0.504), and weak correlations with *v*_*e*_ (R^2^ = 0.150), *v*_*p*_ (R^2^ = 0.077), *PS* (R^2^ = 0.117),* K*^*trans*^ (R^2^ = 0.088) and whole tumor volume (R^2^ = 0.174). In the treatment study, the *k*_*io*_ of the treated group was significantly lower than the control group one day after bevacizumab treatment and decreased significantly after 5FU treatment compared to the baseline. This study results support the feasibility of measuring *k*_*io*_ using the two flip-angle DCE-MRI approach in cancer imaging.

## Introduction

The water efflux rate constant (*k*_*io*_) is a parameter of interest for oncology studies since cellular water exchange may reflect pathophysiological conditions of the cancer cells^1^. Cellular-interstitial water exchange may be a sensitive indicator of cellular energy turnover, as the water exchange depends on cell membrane ion-pump activities, a measure of glycolysis and mitochondrial respiration^2,3^. Increased expression of transmembrane water channels, aquaporins (AQPs), and subsequent increase of cell membrane permeability are associated with angiogenesis, proliferation and metastatic potential in cancer^4^. Estimation of *k*_*io*_ from pharmacokinetic modeling of dynamic contrast-enhanced (DCE) MRI has shown sensitivity to these different mechanisms of cellular water exchange^1^. Furthermore, several cancer studies have demonstrated the feasibility of using *k*_*io*_ for tumor selection and grading^5–7^ and predicting treatment response^1,8–10^ including long term survivability^11^.

However, achieving accurate and reliable estimation of *k*_*io*_ from DCE-MRI data has proven to be non-trivial due to the sensitivity of *k*_*io*_ estimation to various experimental conditions, such as MRI scan parameters, arterial input function (AIF) and selection of contrast kinetic models^12–14^. It was also reported that the degree of water exchange effect on DCE-MRI data depends on scan protocols including the flip angle^12,15^. This flip angle dependency was leveraged in a previous study^16^ to improve the accuracy of *k*_*io*_ estimation by actively encoding the water exchange effect in the washout phase of the DCE scan with a series of multiple flip angles, i.e., with different sensitivities to cellular water exchange. As the weighting of the water exchange effect changes with the different flip angle segments, one set of contrast kinetic parameters cannot adequately model the data without including the *k*_*io*_ term.

The present study aims to evaluate the robustness and significance of the *k*_*io*_ parameter estimated using the two flip-angle DCE-MRI approach in GL261 murine glioma model. The repeatability of contrast kinetic parameter measurement and *k*_*io*_ was assessed by a test–retest DCE-MRI experiment. The association of *k*_*io*_ with cellular metabolism was investigated through consecutive DCE-MRI and ^18^F-fluorodeoxyglucose (FDG)—Positron Emission Tomography (PET) scans. Finally, we assessed the feasibility of *k*_*io*_ in evaluating tumor progression and response to chemotherapy.

## Methods

### Animal model

Six to eight-week-old C57BL6 mice (*n* = 24; female) with GL261 mouse glioma models were included in this study for repeatability measurement (n = 7), comparison with FDG-PET (n = 7), and assessment of treatment response (n = 10), as described below in detail. For this study to assess the feasibility of using the proposed two-flip angle DCE-MRI method, no power calculation was used to determine the sample size. The mice were inoculated with 1 × 10^5^ GL261 mouse glioma cells suspended in 4μL of phosphate buffered saline (PBS) solution using a Hamilton syringe for stereotactic intracranial injection into the subcortex (2.5 mm depth). The inclusion criterion was to have successful tumor growth observed by MRI between day 15 and 26 after tumor inoculation. The exclusion criterion was to have any sign of pain and distress. All the mice included in this study meet these criteria. All animals were treated in strict accordance with the National Institutes of Health Guide for the Care and Use of Laboratory Animals, and this study was approved by the institutional animal care of use committees (IACUC) at Weill Cornell Medical College and New York University School of Medicine and reported in accordance with ARRIVE guidelines.

### DCE-MRI data acquisition

MRI scans were performed on a Bruker 7 T micro-MRI system equipped with an 80-mm transmit-only birdcage coil and a ^1^H four-channel phased array cryogenically cooled receive-only mouse brain MRI coil. DCE-MRI image acquisition was performed using the 3D ultra-short echo-time (UTE) pulse sequence (*TR* = 4 ms and *TE* = 0.028 ms) with 3D Golden angle Radial Sparse Parallel (GRASP) MRI method to achieve an isotropic spatial resolution and to minimize the *T*_2_^*^ effect. The pulse sequence was continuously run to acquire 154,080 spokes for 10 min and 13 s^16^. The two flip-angle DCE-MRI method was implemented by using a series of three flip angle segments (8°-25°-8°) with 51,360 spokes per flip angle segment (image matrix size = 128 × 128 × 128, field of view = 20 × 20 × 20 mm^3^, and the spatial resolution = 0.156 × 0.156 × 0.156 mm^3^). The segments with a low flip angle (8°) are expected to have larger effects of the cellular-interstitial water exchange than the one with a high flip angle (25°)^16^. A bolus of gadolinium-based contrast agent (GBCA) in saline at the dose of 0.1 mmol/kg was injected through a tail vein catheter, starting 60 s after the start of data acquisition; gadoxetate disodium (Eovist, Bayer) was used for the repeatability measurement and gadobutrol (Gadavist, Bayer) for comparison with FDG-PET and assessment of treatment response, assuming these two GBCAs work similarly as non-specific contrast agents in tumor. Prior to each DCE-MRI experiment, a 3D *T*_*1*_ map with the same isotropic high resolution was obtained using the same 3D-UTE-GRASP sequence with variable flip angles (8°-2°-12°, 12,776 spokes for each flip angle, total acquisition time = 153 s)^16^.

### DCE-MRI data analysis

Image reconstruction was conducted using the joint compressed sensing and parallel imaging reconstruction method based on the 3D-UTE-GRASP algorithm^17^ in order to achieve a temporal frame resolution *T* = 5 s/frame. Arterial input function (AIF) was obtained from the early enhancing vascular voxels following the Principal Component Analysis (PCA) method used in our previous study^18^. The *T*_*1*_-weighted images were used to manually segment tumors. The independently measured pre-contrast *T*_*1*_ map was used to convert the DCE-MRI signal intensity data to GBCA concentration data with the assumption of the tissue relaxivity (*r*_*1*_ = 5.1 s^−1^ mmol^−1^ for gadoxetate and 3.3 s^−1^ mmol^−1^ for gadobutrol) and a homogeneous radiofrequency transmit field for the volume coil (i.e., ${B}_{1}^{+}=1$). Pharmacokinetic model analysis was carried out for the whole tumor with the Two Compartment Exchange Model (TCM)^19^ for GBCA combined with the Three Site Two Exchange Model (3S2X)^15,20,21^ for water exchange in order to estimate five parameters: interstitial space volume fraction (*v*_*e*_), vascular space volume fraction (*v*_*p*_), blood flow (*F*_*p*_), permeability surface area product (*PS*), and water efflux rate constant (*k*_*io*_). Transfer constant (*K*^*trans*^) was calculated from *PS* and *F*_*p*_ (*K*^*trans*^ = *[1-exp(-PS/F*_*p*_*)] Fp*). In addition to *k*_*io*_ which reflects the efflux rate constant from the intracellular compartment, we also included a transcytolemmal exchange rate constant defined as ${k}_{ex}={k}_{io}/{v}_{e}={k}_{oi}/{v}_{i}$ (*i.e*. ${\tau }_{ex}={v}_{e}{\tau }_{i}={v}_{i}{\tau }_{e}$), which takes into account the compartmental volume fractions^22–24^. We assumed that the water fraction *f*_*w*_ is 1 in all compartments for simplicity; while the conventional assumption is *f*_*w*_ = 0.8 for the normal brain, it is not trivial to find appropriate *f*_*w*_ values for the intra- and extracellular compartments in various locations of heterogeneous tumors like the ones included in this study.

### Test–retest repeatability

**Figure 1:**
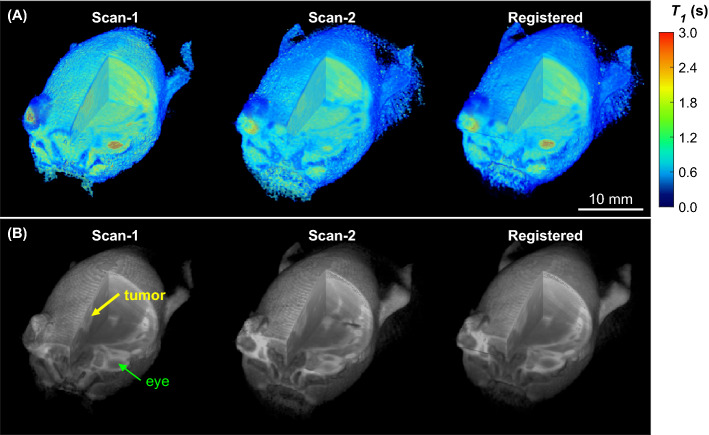
Volume rendered images of the two consecutive DCE-MRI scans of a same mouse with an interval of 30 min. A cutout is made to show the cross-sections through a tumor (yellow arrow) and an eye (green arrow). The images of Scan-2 were registered to those of Scan-1 as shown in the right column. (**A**) 3D T1 map generated from 3D-UTE-GRASP images with three flip angles. (**B**) One of DCE-MR images, reconstructed with a temporal resolution of 5 s/frame, corresponding to 120 s after contrast injection.

The repeatability of contrast kinetic parameter and *k*_*io*_ estimation was measured in the C57BL6 mice with GL261 intracranial tumors (n = 7) between day 15 and day 26 after tumor inoculation in the brain. For each mouse, the DCE-MRI scan with two flip angles was conducted twice with an interval of 30 min between the end of the first DCE-MRI scan and the beginning of the second scan with the *T*_1_ mapping followed by the second DCE-MRI. Upon the completion of the first scan, the mouse was moved out of the magnet and then repositioned in the magnet for the second scan. Each DCE-MRI scan included a GBCA injection at the dose of 0.1 mmol/kg. To align the tumor voxels between the first and the second DCE-MRI scans, the images of the second DCE-MRI scan (both *T*_*1*_ map and DCE-MRI) were registered to the images of the first DCE-MRI by applying rigid body transformation through minimization of the sum of mean squared differences between the two 3D image datasets (Fig. 1).

The contrast kinetic parameters and *k*_*io*_ of tumor voxels from the two scans were compared at the voxel level using the Wilcoxon signed rank test. The contrast kinetic parameter and *k*_*io*_ maps of the test and re-test scans were also compared in terms of texture features extracted using the open-source Python package PyRadiomics^25^. We included 93 features; First Order Histogram Features (n = 18 features), Gray Level Co-occurrence Matrix (GLCM; n = 24), Gray Level Dependence Matrix (GLDM; n = 14), Gray Level Run Length Matrix (GLRLM; n = 16), Gray Level Size Zone Matrix (GLSZM; n = 16), and Neighboring Gray Tone Difference Matrix (NGTDM; n = 5). In addition to the contrast kinetic parameter and *k*_*io*_ maps, the last frame of the DCE-MR images was included as delayed contrast enhanced images for extracting texture features. Discretization of the dynamic ranges of the contrast enhanced images and parameter maps was performed using a fixed bin width determined by the Freedman-Diaconis rule: *W* = *2 (IQR) N*^*-1/3*^, where *IQR* is the interquartile range and *N* is the number of pixels^26^. The default PyRadiomics configuration was used for all other texture feature extraction settings (see http://pyradiomics.readthedocs.io for further information). Statistical analysis of texture feature differences between two images was performed using the paired Student’s t-test. To keep the family-wise error rate to 0.05 for each parameter, we used the significance level for a single hypothesis test of 0.0006.

### Comparison of DCE-MRI with FDG-PET

Previous studies have shown that the water exchange across the cell plasma membrane is associated with the expression of the ATP-dependent ion channels^2,3^. The goal of this experiment is to assess the association between *k*_*io*_ as a measure of transcytolemmal water exchange and FDG uptake as a marker for cellular metabolism. Six to eight-week-old C57BL6 mice (*n* = 7) with GL261 mouse glioma xenograft models were used for this experiment. *k*_*io*_ was estimated using the two flip-angle DCE-MRI method. To ensure that the well perfused voxels with sufficient GBCA extravasation were selected for reliable measurement of *k*_*io*_, the top 10% of voxels with the highest signal enhancement ratio were selected for analysis^12,20,27–29^. This selection was used particularly due to low contrast enhancement of the glioma model used in this study (average *K*^*trans*^ < 0.1 min^-1^ for all tumors included for this comparison as shown in Fig. 5). We assumed the *k*_*io*_ measured from the most enhancing part of the tumor is representative of the whole tumor for the comparison with the FDG-PET. The DCE-MRI scan was immediately followed by ^18^F-FDG-PET scan per animal. ^18^F-FDG PET and computed tomography (CT) scans were performed on an Inveon small-animal hybrid PET/CT scanner (Siemens Medical Solutions). The mice were administered an ^18^F-FDG bolus (6.45–11.1 MBq/0.1 mL) via tail vein injection. The PET data collected for 5 min after 55 min post injection were used to generate the standardized uptake value (SUV) maps. SUV was calculated as: [decay corrected activity (Bq/mL)]/[animal weight (g)]/[injected dose (Bq)]. Whole tumors were manually segmented from co-registered PET/CT images. Since the low spatial resolution of PET images (1.4 mm isotropic) is not adequate for voxel-level analysis, the mean SUV of the whole tumor was used for the comparison with DCE-MRI.

### Assessment of treatment response

Two groups of six to eight-week-old C57BL6 mice (*n* = 5 per group) with GL261 mouse glioma model were used to investigate the feasibility of using the two flip-angle DCE-MRI approach for longitudinal assessment of treatment response. For the treatment group, pre-treatment DCE-MRI (Pre-Tx) was immediately followed by an intraperitoneal (IP) injection of bevacizumab (Avastin, Genentech) at 10 mg/kg. After 24 h, mice were scanned again (Tx-1) and subsequently given an IP injection of fluorouracil (5FU, Fressenius Kabi) at 80 mg/kg. A second dose of 5FU (80 mg/kg) was given after 48 h. Post-treatment DCE-MRI (Tx-2) was conducted 24 h after the second 5FU treatment (i.e. 4 days after the pre-treatment DCE-MRI). The control group was treated with sodium chloride solution (10 ml/kg) and imaged at the same time points as the treatment group (Supplementary Fig. 1). Control and treated groups were randomly selected by a co-author (SGK) who was blinded to the animal status and images at the selection. Blinding of treatment group was not used in this feasibility study with a small number of animals. We assumed the benefit of blinding would be insignificant since the data collection and analysis were conducted with a same scan protocol and data processing pipeline.

## Results

### Repeatability

**Figure 2:**
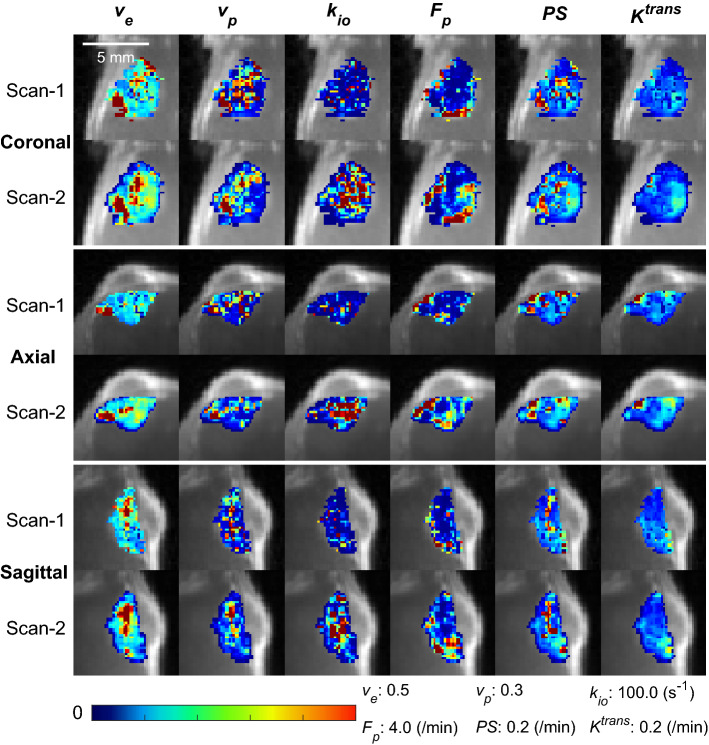
Contrast kinetic parameter and *k*_*io*_ maps of a GL261 tumor estimated using the 3D-UTE-GRASP DCE-MRI data acquired with the proposed two flip-angle method and an isotropic 3D resolution. This is a representative case from the test–retest study (n = 7) in which each mouse was scanned twice consecutively with an interval of 30 min. The whole tumor parameter maps of the Scan-2 are from the Scan-2 images registered to the Scan-1 images as shown in Fig. 1.

**Figure 3:**
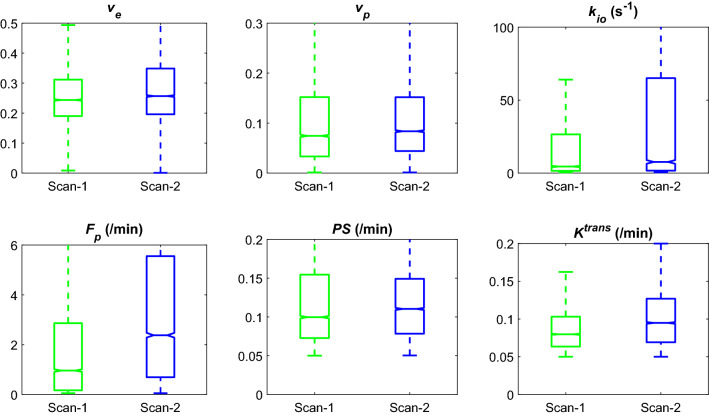
Comparison of whole tumor contrast kinetic parameters and *k*_*io*_ obtained from test–retest scans. All voxels in the tumors (n = 7) are pooled together for the box-whisker plots. There is no significant difference between the two scans in terms of the median values of parameters, except *K*^*trans*^ (P = 0.047). *K*^*trans*^ values were calculated from the *F*_*p*_ and *PS* values for individual voxels.

The DCE-MRI scans were successfully conducted twice for a group of mice (n = 7) included in this experiment. In all mice, the whole tumor contrast kinetic parameter maps and *k*_*io*_ from the two scans of a same tumor appear similar to each other (Fig. 2). Among the estimated parameters, the volume fraction measures, *v*_*e*_ and *v*_*p*_, show strong correlation between the two scans (Pearson correlation coefficient r = 0.61 for *v*_*e*_ and 0.70 for *v*_*p*_) and no significant difference between them (P = 0.375 for *v*_*e*_ and 0.467 for *v*_*p*_; Wilcoxon signed rank test) (Fig. 3). The vascular parameters, *PS, F*_*p*_, and *K*^*trans*^, appear to be greater in the second scan than in the first scan: 41.8% greater for PS (P = 0.109), 261.5% greater for *F*_*p*_ (P = 0.078), and 43.2% greater for *K*^*trans*^ (P = 0.047) in terms of the median values. In contrast, *k*_*io*_ values increased between the first and second scan by 45.2% of the median value (P = 0.156). Of the 93 histogram and texture features extracted from the parameter maps, 9 features in *k*_*io*_ maps and one feature in *v*_*e*_ and *v*_*p*_ maps were found to be significantly different (Supplemental Fig. S1).

### k_io_ correlates with SUV

**Figure 4:**
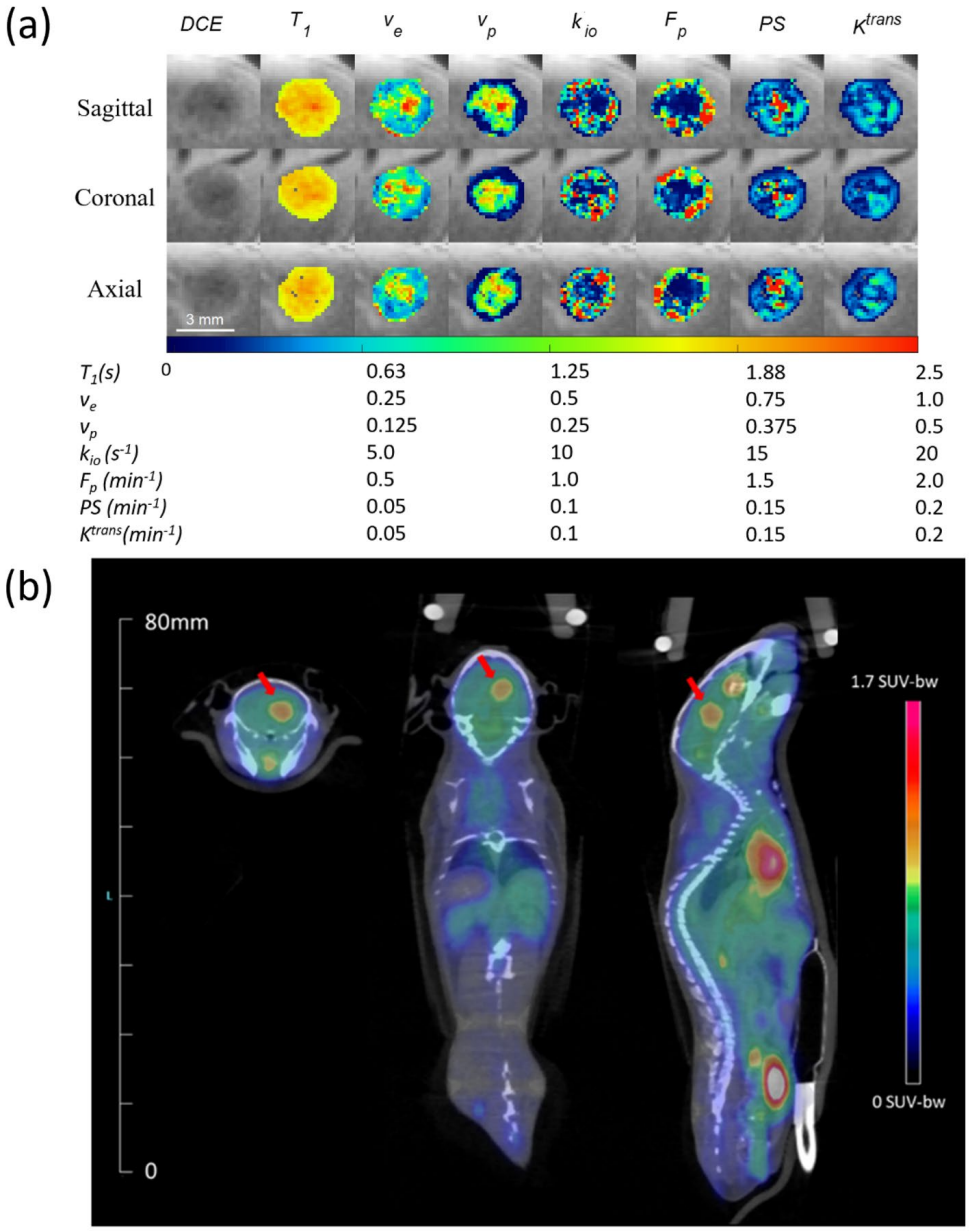
A representative case of a mouse with GL261 tumor scanned using DCE-MRI (**a**), immediately followed by ^18^F-FDG-PET/CT (**b**). DCE-MRI scan was conducted using a high spatial resolution (0.156 × 0.156 × 0.156 mm^3^) for the brain, whereas FDG-PET had a relatively lower spatial resolution (1.4 × 1.4 × 1.4 mm^3^) covering the whole body. The heterogeneity of the tumor observed in DCE-MRI (**a**) is not clearly shown in the SUV map of the tumor (arrows) with a lower spatial resolution, overlaid on the CT image (**b**). The SUV map (**b**) was generated by the vendor software which may include some image processing for the display, whereas the DCE-MRI parameter maps (**a**) were shown without any interpolation or smoothing.

**Figure 5:**
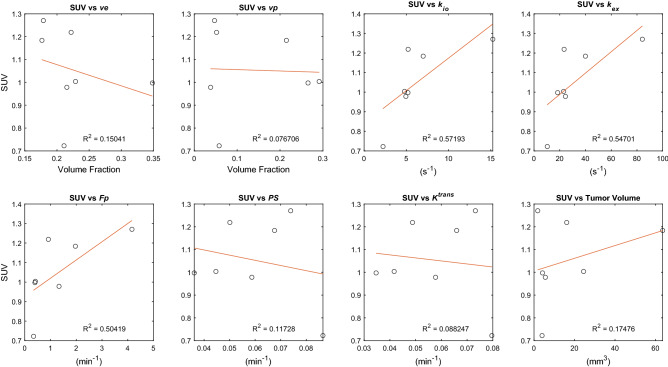
Scatter plots to assess the association between the contrast kinetic parameters and *k*_*io*_ estimated from top ten percent of enhancing voxels from DCE-MRI and whole tumor standardized uptake values (SUV) from FDG-PET. Data used in these plots are the median values of individual tumors (n = 7). The red lines are linear regression lines with the R^2^ values shown in the plots.

The correlation between the contrast kinetic parameters (including *k*_*io*_) and SUV was assessed using a separate group of mice (n = 7) which had DCE-MRI scan immediately followed by FDG-PET. The 3D maps of contrast kinetic parameters and *k*_*io*_ have a relatively high spatial resolution that shows heterogeneous patterns of the tumor tissue, which cannot be appreciated in the SUV maps of FDG-PET with a lower spatial resolution (Fig. 4). The median values of the contrast kinetic parameters from the top ten percent enhancing voxels are compared with the mean SUV values (Fig. 5). The scatter plots in Fig. 5 show that SUV has a positive correlation with *k*_*io*_ (R^2^ = 0.572), *k*_*ex*_ (R^2^ = 0.547), a positive correlation with *F*_*p*_ (R^2^ = 0.504), and weak correlations with *v*_*e*_ (R^2^ = 0.150), *v*_*p*_ (R^2^ = 0.077), *PS* (R^2^ = 0.117), *K*^*trans*^ (R^2^ = 0.088) and whole tumor volume (R^2^ = 0.174). Whole tumor SUV compared to whole tumor contrast kinetic parameter and *k*_*io*_ maps demonstrated strong correlations with *PS* (R^2^ = 0.693) and* K*^*trans*^ (R^2^ = 0.651) and weak correlations with all other parameters (Supplemental Fig. 2).

### k_io_ is a sensitive marker for treatment response

**Figure 6:**
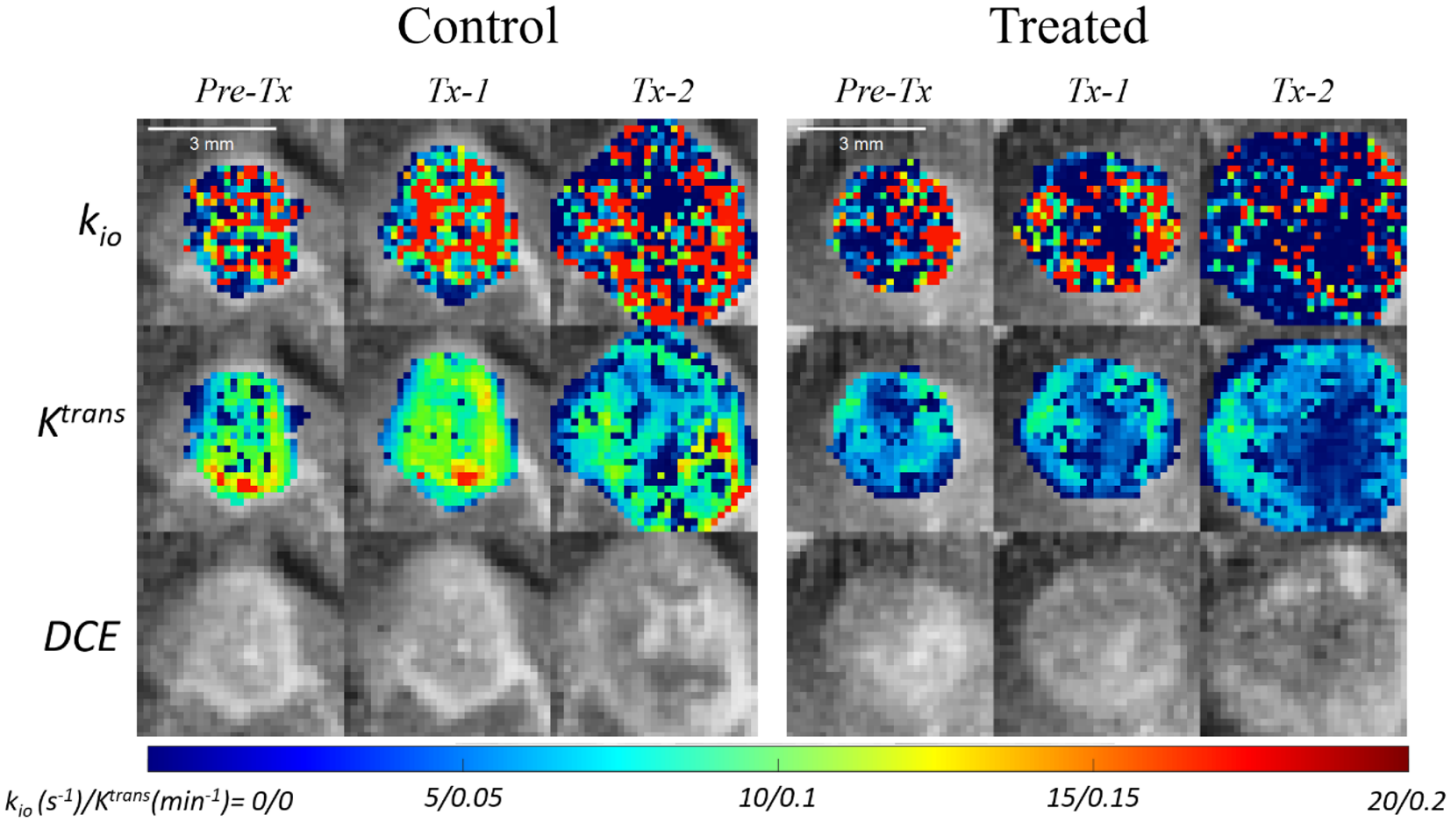
Representative *k*_*io*_ and *K*^*trans*^ parameter maps and the last frame of DCE-MR images of a mouse from the control group (left) and one from the treated group (right). The parameter maps are overlayed on the last frame of DCE-MR images (DCE). In both cases, it can be observed that the tumors grew aggressively within 4 days between Pre-tx and Tx-2.

**Figure 7:**
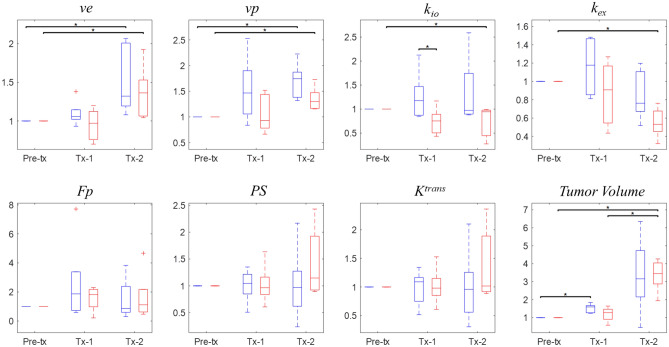
Normalized whole tumor contrast kinetic parameter and *k*_*io*_ median values for the control (blue, left) and treated (red, right) cohorts for pre-treatment (Pre-tx), 24 h post bevacizumab (Tx-1) and post two doses of fluorouracil (Tx-2) time points. Normalization was done by dividing each parameter by the Pre-tx values of the individual tumor. * denotes a statistical significance (P < 0.05) by the Wilcoxon rank sum test.

The intracranial tumors in this study were growing aggressively; the tumors in both control and treated groups approximately tripled in volumes within the 4 days, from 3.10 ± 6.33 uL to 9.88 ± 26.73 uL, between the pre-treatment and Tx-2 (Figs. 6 and 7). While the volume did not show a significant difference between the control and treated groups at any time point, the *k*_*io*_ normalized to the baseline value showed a significant difference (P = 0.032) between the two groups at Tx-1. The normalized *k*_*io*_ and *k*_*ex*_ of the control group did not change significantly in the two imaging time points, while those of the treated group decreased significantly (P = 0.008 for both *k*_*io*_ and *k*_*ex*_) at Tx-2. This pattern of change in *k*_*io*_ is observed more clearly in the tumor rims as shown in Fig. 6. The normalized *v*_*e*_ and *v*_*p*_ increased significantly (P = 0.008) between Pre-Tx and Tx-2 in both groups. In this study with a small number of animals, no significant difference between the two groups was observed in the contrast kinetic parameters and *k*_*io*_ without normalization to the baseline values (Table 1).

**Table 1:** Whole tumor contrast kinetic parameters and *k*_*io*_ (median (IQR)) measured at pre-treatment (Pre-tx), 24 h post bevacizumab (Tx-2) and post two doses of fluorouracil (Tx-2).

		*v*_*e*_	*v*_*p*_	*k*_*io*_* (s*^*−1*^*)*	*F*_*p*_* (min*^*−1*^*)*	*PS (min*^*−1*^*)*	*K*^*trans*^*(min*^*−1*^*)*
Control	Pre-Tx	0.171 (0.091)	0.037 (0.082)	0.751 (3.970)	0.357 (0.826)	0.059 (0.068)	0.050 (0.050)
	Tx-1	0.180 (0.073)	0.042 (0.087)	1.335 (3.395)	0.694 (0.906)	0.069 (0.067)	0.049 (0.046)
	Tx-2	0.213 (0.074)	0.083 (0.130)	1.626 (3.020)	0.505 (0.333)	0.028 (0.035)	0.026 (0.029)
Treated	Pre-Tx	0.149 (0.050)	0.026 (0.022)	2.250 (1.600)	0.471 (1.057)	0.014 (0.017)	0.014 (0.014)
	Tx-1	0.120 (0.096)	0.025 (0.021)	1.251 (0.996)	0.586 (0.595)	0.014 (0.010)	0.013 (0.008)
	Tx-2	0.187 (0.062)	0.034 (0.030)	0.760 (0.486)	0.713 (0.787)	0.021 (0.016)	0.020 (0.012)

The parameters normalized to the individual baseline values are shown in the plots of Fig. 7.

## Discussion

Quantitative tissue microenvironment parameters measured from DCE-MRI may non-invasively capture the heterogenous properties of a tumor and their changes during tumor progresses and treatment. However, the adoption of these parameters in routine clinical imaging remains challenging and requires more work to establish the repeatability and reproducibility of the DCE-MRI data acquisition and analysis method^30^. Among the parameters of interest, the water efflux rate constant, *k*_*io*_, is a unique parameter with potential as a biomarker for cellular energy turnover, but has proven difficult to measure^12,13^. The recently proposed method of using a multiple flip angle approach to actively encode the water exchange effect in the washout phase of the DCE-MRI scan, has demonstrated potential for improving the accuracy and precision of *k*_*io*_ estimation. In this present study, we further evaluated the multiple flip-angle method in terms of repeatability, correlation with SUV and sensitivity to treatment response.

We found that the compartmental volume fractions (*v*_*e*_ and *v*_*p*_) remained consistent between the test and retest scans while the vascular functional measures (*F*_*p*_ and *PS)* and *k*_*io*_ showed noticeable changes, probably due to physiological changes of the tumor between the two scans. It is not trivial to measure repeatability of DCE-MRI parameters as the subject must fully clear the GBCA before a retest may be performed. On the other hand, aggressive tumors could have continuous changes in their volume and properties as observed in this study with GL261. For our study, hence, we performed a test–retest with a limited time interval of 30 min for GBCA to clear from the body as much as possible before repeating the DCE-MRI scan. We conducted *T*_1_ mapping prior to each DCE-MRI scan such that the potential bias of remaining contrast agent in the tissue can be minimized in the contrast kinetic model analysis of the second data set.

Between the test and retest scans, we observed negligible changes in the estimated compartmental volume fraction parameters *v*_*e*_ and *v*_*p*_. This is a reasonable outcome as we would not expect the cellular volumetric properties of the tumors to change substantially over the short duration of the experiment. In contrast, we observed noticeable increases in perfusion parameters (*F*_*p*_, *PS,* and *K*^*trans*^) and an increase in *k*_*io*_. These changes from the first to the second scan may be explained by physiological changes induced by prolonged exposure to isoflurane altering blood flow in the mouse and tumor metabolism. Li et al.^31^ demonstrated that cerebral blood flow in macaques increased in a dose dependent manner with isoflurane concentration. While isoflurane levels were maintained at low maintenance dosage (1–1.5% in air) throughout the experiment, prolonged exposure likely resulted in increased blood flow due to vasodilation. Furthermore, isoflurane has been shown to upregulate hypoxia-inducible factor 1-alpha (HIF1A) and vascular endothelial growth factor (VEGF) in cancer^32,33^. In vitro studies of ovarian cancer cells found that exposure to isoflurane increased angiogenesis, cell proliferation, and migration associated with increased levels of insulin-like growth factor 1 and VEGF^34^. One of the most prominent adaptations in hypoxic cells is increased glucose uptake and expression of glutamine transporters, to promote glucose catabolism^35^. The increase in glucose metabolism would lead to an increase in water exchange, i.e. an increase in *k*_*io*_. Hence, although the results of our study did not show a strong repeatability of *k*_*io*_, they may suggest the sensitivity of *k*_*io*_ to the metabolic status of the cancer cells.

To better understand and probe the relationship of cellular water exchange with cellular metabolism, we compared *k*_*io*_ from DCE-MRI with SUV from ^18^F-FDG-PET. SUV is widely used as a semi-quantitative measure of glucose uptake and phosphorylation in tissue, an indicator of cellular metabolic activity. High SUV values are known to be associated with poor prognosis^36^. The correlation between *k*_*io*_ and SUV observed in this study further indicates *k*_*io*_ as a sensitive parameter for cellular metabolic activity. This observation is also in line with previous studies that showed the cellular water exchange is associated with Na + /K + ATPase activity and ATP-dependent ion channels in the plasma membrane^2,37,38^. The weak correlation between tumor volume and SUV suggests that the relationship between *k*_*io*_ and SUV is more likely driven by energy turnover than the bias of larger tumors acting as an FDG sink.

Several studies have demonstrated the feasibility of using *k*_*io*_ for monitoring and predicting tumor response to treatment and long term survivability^1,6,8,9,11^. Our present study explored the feasibility of using the multiple flip angle approach to estimate *k*_*io*_ during treatment of murine glioma using a combination therapy of bevacizumab, a monoclonal antibody against VEGF, and fluorouracil (5FU), a cytotoxic chemotherapeutic agent. Bevacizumab is often used in combination with other cytotoxic therapies as it could be used to normalize tumor vasculature, improving delivery of the secondary chemotherapeutic agent^39,40^. The effect of this drug combination appears to be captured well by our study, as we observed a decrease both in water exchange and *v*_*p*_ in the treated group, despite a persistent increase in tumor volume in both groups. Often, responding tumors do not demonstrate an immediate change in volume, or even grow after the first several treatments. This highlights the importance of biomarkers which may detect tumor response prior to volume reduction. Further study is warranted to establish these DCE-MRI measures as biomarkers to simultaneously measure the anti-angiogenic and cytotoxic effect of a combination therapy.

Limitations of our study includes small cohort sizes used in the individual experiments. While the statistical power is limited by the small cohort size, our study results demonstrate that the multiple flip angle method of DCE-MRI can be used as a robust way to measure *k*_*io*_ in cancer imaging studies and can serve as the pilot data to design future studies with larger cohorts. The present study is also limited to single tumor model. Future studies can also include pathological measurements of transmembrane water channels as well as other proteins related to cellular metabolism, such as mitochondria pyruvate carrier, monocarboxylate transport, lactate dehydrogenase A, lactate dehydrogenase B and pyruvate dehydrogenase^41^.

In this study, we found that contrast kinetic parameters and *k*_*io*_ of tumor can be estimated from a multiple flip-angle DCE-MRI data. We have demonstrated that *k*_*io*_ has a strong correlation with the SUV from ^18^F-FDG PET, suggesting the feasibility of using *k*_*io*_ as an imaging marker for cellular metabolic activity without using a radioactive contrast agent. The results of this study also support the potential of *k*_*io*_ to discern tumors responding to a vascular normalization treatment and cytotoxic chemotherapeutics. Further study with a larger cohort is warranted to establish the cellular water exchange in cancer metabolism and its changes during progression and treatment.

## Supplementary Information

Supplementary Figures.

## Data Availability

The datasets used and/or analyzed during the current study available from the corresponding author on reasonable request.
