# Supplementary material for: Evaluation of cellular water exchange in a mouse glioma model using dynamic contrast-enhanced MRI with two flip angles
Source: Sci Rep. 2023 Feb 21;13:3007. doi: 10.1038/s41598-023-29991-1 (PMC9945648; doi:10.1038/s41598-023-29991-1)
Supplement: Supplementary file 1 — Supplementary Figures. [file 41598_2023_29991_MOESM1_ESM.pdf]

Evaluation of cellular water exchange in a mouse glioma model using Dynamic Contrast-Enhanced MRI with two flip angles

Karl Kiser[1], Jin Zhang[1], Ayesha Bharadwaj Das[1], James A. Tranos[2], Youssef Zaim Wadghiri[2], and S. Gene Kim[1]*

[1]Department of Radiology, Weill Cornell Medical College, New York, NY, United States

[2]Center for Biomedical Imaging (CBI), Center for Advanced Imaging Innovation and Research (CAI^{2}R), Department of Radiology, NYU Grossman School of Medicine, New York, NY, USA

Send correspondence to:

Karl Kiser

E-mail: karl.j.kiser@gmail.com

Tel: (240) 753-8416

12 Pompanuck Way

Cambridge, NY, 12816

Grant sponsor: National Institutes of Health; Grant number: R01CA160620, R01CA219964, and UH3CA22869.

Supplemental Figures

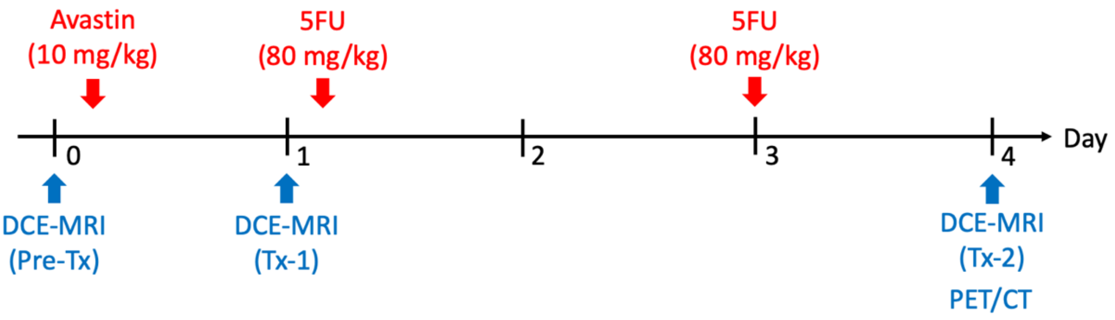

Supplemental Figure S1. Timing diagram for imaging protocol and treatment regime for the assessment of treatment response study. For the treatment group, pre-treatment DCE-MRI (Pre-Tx) was immediately followed by an intraperitoneal (IP) injection of bevacizumab (Avastin, Genentech) at 10 mg/kg. After 24 hours, mice were scanned again (Tx-1) and subsequently given an IP injection of fluorouracil (5FU, Fressenius Kabi) at 80mg/kg. A second dose of 5FU (80mg/kg) was given after 48 hours. Post-treatment DCE-MRI (Tx-2) was conducted 24 hours after the second 5FU treatment (i.e., four days after the pre-treatment DCE-MRI). The control group was treated with sodium chloride solution (10 ml/kg) and imaged at the same time points as the treatment group.

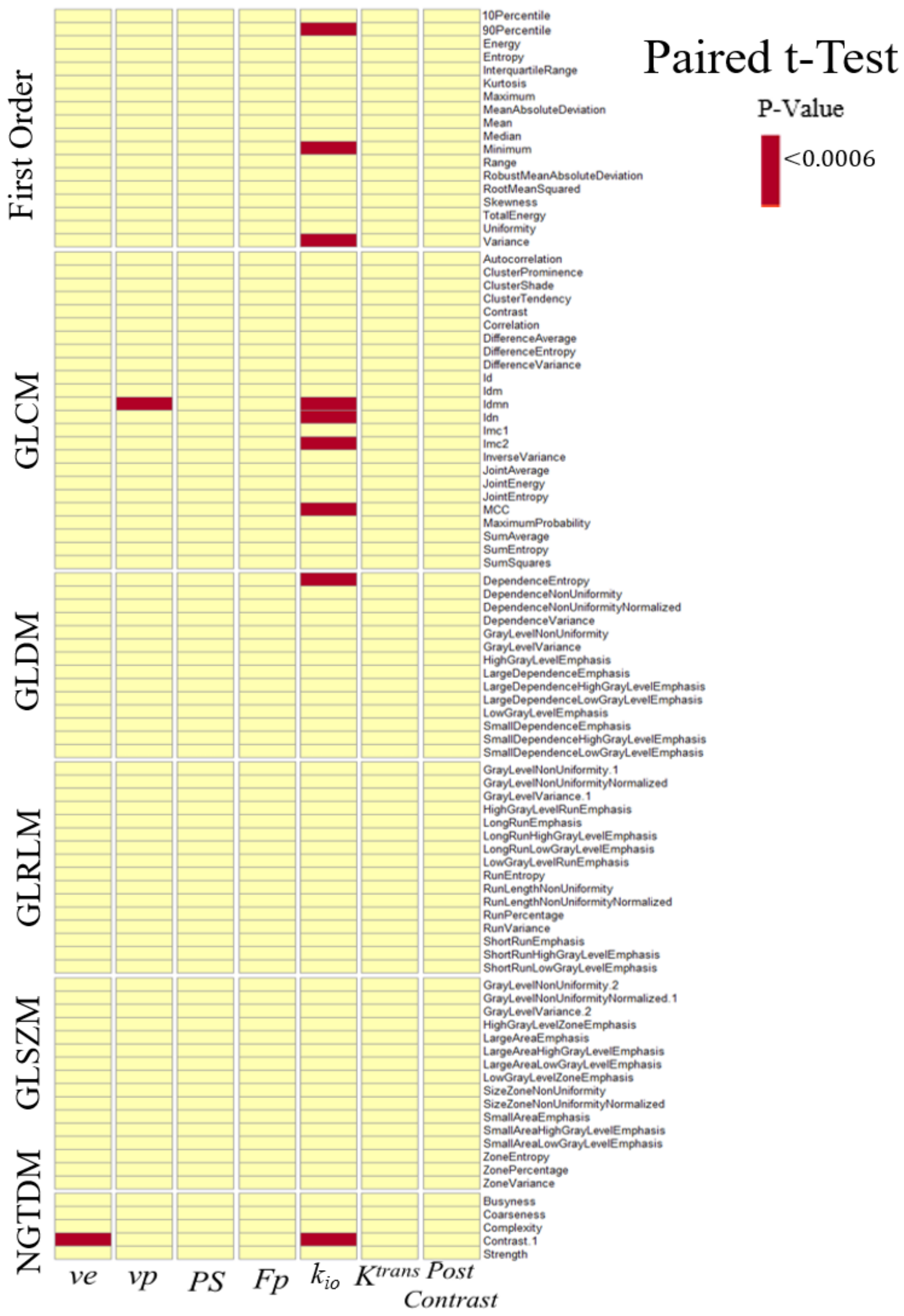

Supplemental Figure S2. Heatmap of the radiomics features with significant difference ($p < 0.0006$, depicted in red) between the control and treated cohorts. Among the 93 histogram and texture features extracted from the parameter maps, 9 k_{io} features and one feature for v_e and v_p maps were found to be significantly different. This heatmap was partially created using R Core Team (2022). R: A language and environment for statistical computing. R Foundation for Statistical Computing, Vienna, Austria. URL https://www.R-project.org/.

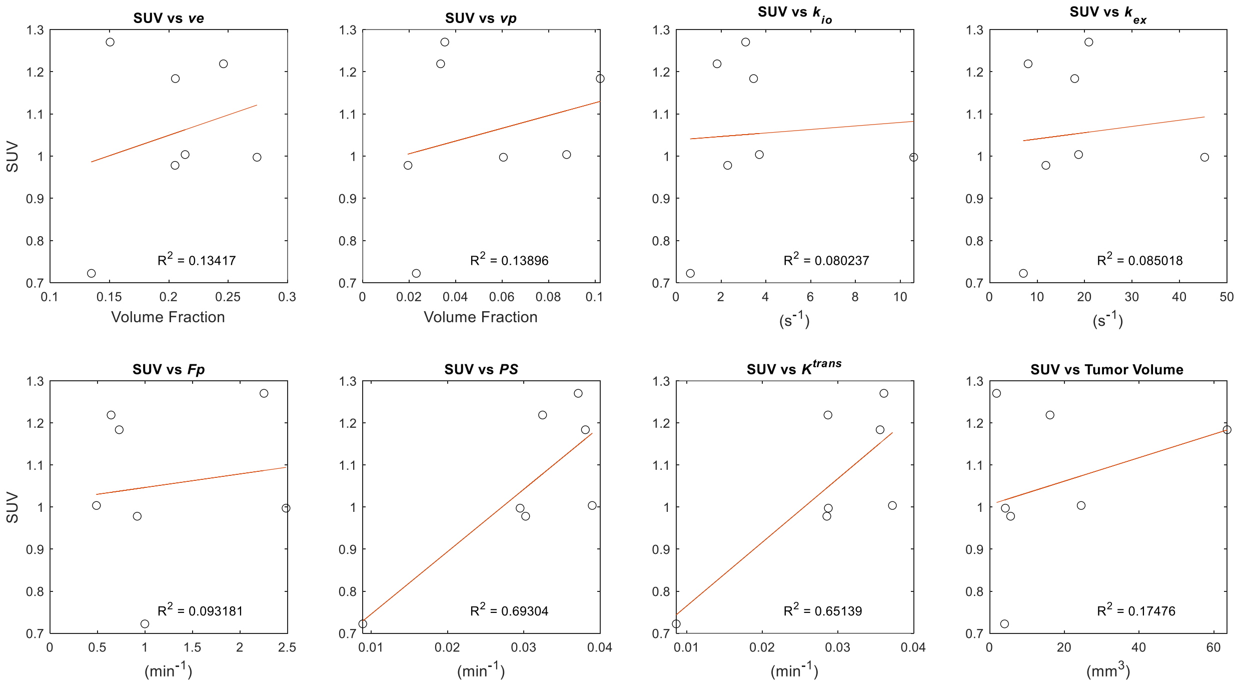

Supplemental Figure S3. Scatter plots to assess the association between the contrast kinetic parameters and k_{io} estimated from whole tumor DCE-MRI and whole tumor standardized uptake values (SUV) from FDG-PET. Data used in these plots are the median values of individual tumors (n=7). The red lines are linear regression lines with the R^2 values shown in the plots.

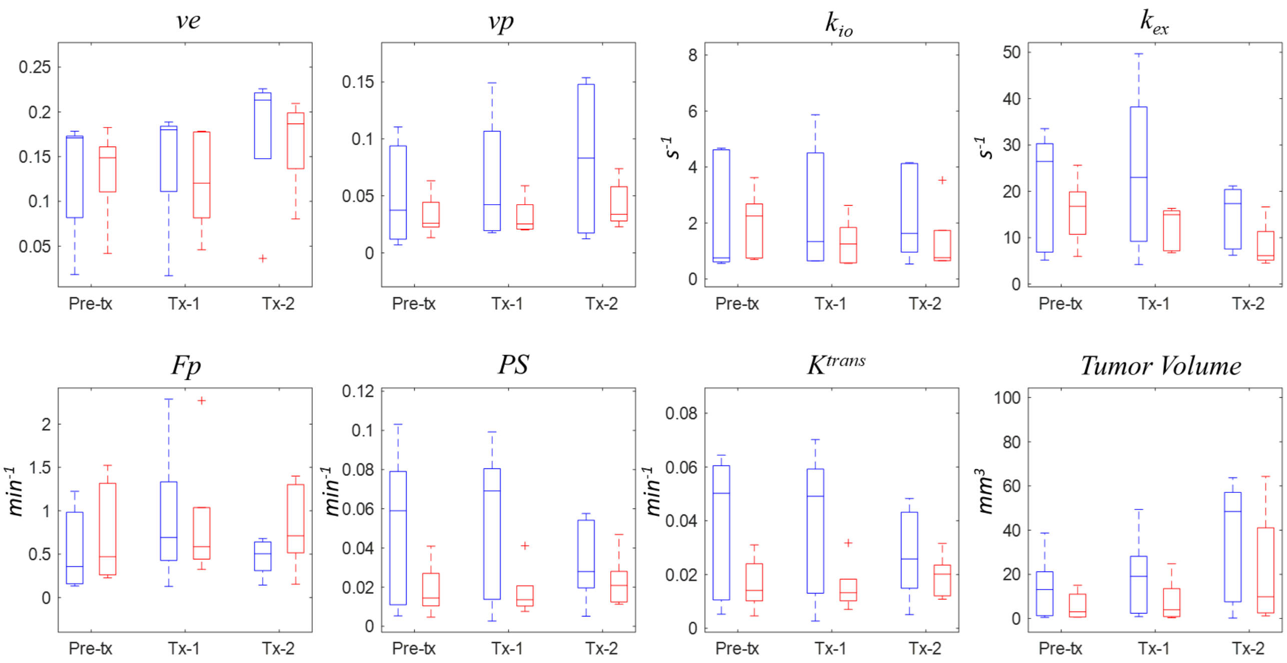

Supplemental Figure S4. Whole tumor contrast kinetic parameter and k_{io} median values for the control (blue, left) and treated (red, right) cohorts for pre-treatment (Pre-tx), 24 hours post bevacizumab (Tx-1) and post two doses of fluorouracil (Tx-2) time points.
